# Tracking the Migraine Cycle Using Visual Tasks

**DOI:** 10.3390/vision4020023

**Published:** 2020-04-30

**Authors:** A.J. Shepherd

**Affiliations:** Department of Psychological Sciences, Birkbeck, University of London, London WC1E 7HX, UK; a.shepherd@bbk.ac.uk; Tel.: +44-20-7631-6212

**Keywords:** migraine, migraine cycle, peri-ictal, vision, motion, orientation

## Abstract

There are a number of reports that perceptual, electrophysiological and imaging measures can track migraine periodicity. As the electrophysiological and imaging research requires specialist equipment, it has few practical applications. This study sought to track changes in performance on four visual tasks over the migraine cycle. Coherence thresholds were measured for two motion and two orientation tasks. The first part of the study confirmed that the data obtained from an online study produced comparable results to those obtained under controlled laboratory conditions. Thirteen migraine with aura, 12 without aura, and 12 healthy controls participated. The second part of the study showed that thresholds for discriminating vertical coherent motion varied with the migraine cycle for a majority of the participants who tested themselves multiple times (four with aura, seven without). Performance improved two days prior to a migraine attack and remained improved for two days afterwards. This outcome is as expected from an extrapolation of earlier electrophysiological research. This research points to the possibility of developing sensitive visual tests that patients can use at home to predict an impending migraine attack and so take steps to try to abort it or, if it is inevitable, to plan their lives around it.

## 1. Introduction

Migraine is a common neurological condition characterised by recurrent, disabling headaches [1]. Many researchers have assessed visual processing in migraine due to the visual disturbances that may precede an attack (the visual aura), the intense sensitivity to light that patients typically experience during an attack (photophobia), and the fact that visual stimuli can trigger attacks in up to 60% of patients (typically, stripes, flicker, and glare) [2,3,4,5,6,7]. Each of these features indicates that migraine is more than a severe headache and that anomalous processing in the visual system is an integral component of the condition. Descriptions of the visual aura suggest that anomalous neural processing can occur throughout the many areas of the cortex involved in visual processing in the occipital, parietal and temporal lobes.

For example, the classic visual aura has been described as a fortification spectra (because it resembles the fortifications or crenellations that define the boundary walls of ancient forts or castles, if viewed from above) or a scintillating scotoma (because there is a zig-zag crescent of activity, scintillating, that grows from central vision to the periphery over approximately half an hour, leaving a blind region in its wake, see [8] for an historical review). 

Early visual cortical areas have a retinotopic organisation, where adjacent points in the visual field are represented in adjacent areas of the cortex. If the visual field is divided vertically at the fovea into right and left halves, the retinotopic map of the left and right hemispheres in the primary visual cortex, V1, corresponds to the right and left visual hemifields, respectively [9]. If the visual field is further divided horizontally into quadrants, the retinotopic maps correspond to separate areas for each quadrant in areas V2 and V3: the maps are discontinuous, or split, at the horizonal meridian. Consequently, adjacent regions of the visual field above and below the horizontal are no longer represented by adjacent areas of the cortex in V2 and V3, yet retinotopy is maintained within each separate quadrant [10,11,12]. The classic visual aura has long been attributed to a wave of activity followed by suppressed neuronal activity (spreading depression) that traverses the primary visual cortex, V1, if restricted to one hemifield [13,14,15,16,17]. By extension, if the aura is restricted to a quadrant, it may be attributed to a wave of activity and subsequent neuronal depression that is probably occurring in V2 or V3. 

Others can see the world as if through running water, or see simpler stars and phosphenes, or they may have pockets of blindness, tunnel vision or double vision. More elaborate distortions can also be experienced such as changes in the relative sizes of parts of images (or of the person’s own body image), where some elements expand and others shrink, which is called the ‘Alice in Wonderland’ syndrome. Indeed, Lewis Carroll’s inspiration for Alice in Wonderland has been credited to the visual aura that he experienced [18]. There are numerous paintings that depict the variety of visual disturbances that can be experienced, which are created by artists inspired by their particular visual aura [19,20]. 

Non-visual somatosensory symptoms can also be experienced as part of an aura (pins and needles, tingling or numbness, often on one side of the face or body), as can language difficulties [1]. Some experience prodromal symptoms, distinct from the aura, such as yawning, mood swings, irritability, and gastrointestinal symptoms [1,21,22]. Elaborate visual experiences, together with somatosensory symptoms and language, indicate anomalous processing in regions anterior to the primary visual cortex that extend into temporal and parietal areas. Double vision, ataxia, and vertigo have been attributed to anomalous activity in the brain stem [1].

The various migraine aura symptoms are part of a migraine attack and typically precede the headache by 30 min or so. There are indications that central neural processing is altered between attacks as well. For example, visual stimuli can trigger migraine on days when the patient is otherwise ostensibly symptom-free [2,4,5,6], which suggests a persistent alteration in neural responses in those areas that process the trigger stimuli. This observation has prompted research to determine whether, between attacks, subtle alterations persist in those areas involved in the migraine aura that could account for visual triggers and, if so, what the nature of these alterations may be. 

There is now ample evidence that several aspects of visual processing differ between migraine and control groups between attacks, such as the perception of contrast, colour, motion, orientation, and visual discomfort reviewed in [23,24,25,26]. Some studies have shown interactions and correlations between these aspects of visual perception and between these aspects and reports of visual migraine triggers [5,6,27,28,29]. Furthermore, performance on a number of visual [30,31,32] and electrophysiological [30,33,34,35,36,37,38] tasks has been reported to vary with attack proximity. Some of these studies assessed proximity with retrospective self-reports and correlated performance with the days reported to have elapsed since the last migraine attack [27,28,29,30,31,32,38]. 

There are also now a number of reports that have tried to track migraine periodicity directly by testing patients sufficiently regularly that they catch changes before, during and after each episode [33,34,35,36,37,38,39,40,41,42,43]; these are reviewed in [44]. There are comparable reports of cyclic fluctuations in imaging studies in migraine, notably in the hypothalamus and brainstem [45,46,47,48]. Furthermore, rapid fluctuations in the activity and structure of brain stem areas have been revealed during noxious stimulation, such as in the 24 h preceding an attack and in the 72 h that follow it [47,48,49]. These peri-ictal variations with the migraine cycle are distinct from reports of structural changes in brain networks that can be observed in thalamic and cortical areas when patients are scanned between and then during attacks [38,50,51,52,53,54]. They are also distinct from reports of differences in resting state activity and connectivity involving the visuospatial system in migraine compared to healthy volunteers [39,53,55,56,57,58,59]. Differences using both electrophysiological and scanning techniques have been reported in areas involved in multisensory integration including the visual, somatosensory, somatomotor, and frontoparietal networks [38,50,51,52,53,54,56,57,59,60,61,62,63]. Any relationships between peri-ictal changes shown in the electrophysiological research and the changes in structure or activity revealed by various scanning techniques, however, have yet to be determined [14]. 

It is notoriously difficult to catch participants in the day or two before a spontaneous migraine attack and have them in the vicinity of a laboratory or hospital to be able to test them in time [46,51]. Indeed, a common complaint is that a migraine happens unexpectedly and those affected live with the anxiety that another attack is imminent. This can have a debilitating effect on a person’s life, impacting social, family, and work activities, and so it also affects those who live or work with them. Another common complaint is that medication only works if taken immediately an attack begins. If patients can learn to recognise when an attack is likely, they could take evasive action to try to abort it (either behaviourally or by taking appropriate medication) or, if it turns out to be inevitable, they could plan around it. Intriguing as the electrophysiological and imaging studies cited above are, they are not practically applicable. Psychophysical visual tests are non-invasive, simpler to administer and, as mentioned above, several have already been shown to be sensitive enough to show differences between migraine and control groups when tested between attacks [4,5,23,24,25,26,27,28,29,30,31,32,41].

This study sought to track changes in performance on four visual tasks over the migraine cycle. Of the many visual tests that have been used to understand group differences between migraine and control groups, the perception of motion and orientation were selected as they have yielded the most consistent patterns of group differences [4,27,28,64,65,66]. Some of these studies have also reported performance to be associated with attack proximity and attack frequency [4,64,66]. 

Coherence thresholds were measured for two motion and two orientation tasks. The oblique effect (OE) was first described by Mach [67] and refers to a reduced ability to make judgements of small orientation differences when lines are oriented at oblique angles (e.g., to discriminate lines oriented at 43° versus 47° left or right of 45°) compared to the “cardinal” orientations, vertical or horizontal (e.g., to discriminate lines oriented 2° left or right of vertical). The OE has been demonstrated for a variety of tasks in addition to those involving oriented lines [68], including coherent motion discrimination [69,70,71,72]. The OE has also been reported to be greater in migraine using oriented gratings (Gabor patches) and virtual lines (an illusory line that can be imagined connecting two widely spaced end-points), which indicated a cortical abnormality in migraine that affects both the primary visual/striate and extrastriate cortical areas [65]. The result with virtual lines suggested that a global shape task, with shape components oriented vertically or obliquely, may be sufficiently sensitive to track changes in orientation discrimination over the migraine cycle. Therefore, cardinal and oblique orientations were included in the present study in tasks involving lines and coherent motion. 

Two sets of investigations were performed: first, using data at one time point to check for overall differences between migraine and control groups (‘Group study’); second, using data from consecutive test sessions to determine whether performance on each task varied with the migraine cycle (‘Longitudinal study’). 

Group study: Based on previous studies on motion [73], it was hypothesised that coherence thresholds for vertical motion (Task 1) would be higher in the migraine than in the control group, in-between attacks. Tasks 2–4 were novel but, extrapolating from Task 1, coherence thresholds were again predicted to be higher in the migraine group than in the control group in-between attacks. Coherence thresholds for oblique motion directions (Task 2) may be greater than those for vertical (after [69]). Coherence thresholds for oriented lines should be greater for lines with oblique orientations compared to horizontal or vertical (after [68]), and the difference may be greater in migraine (after [65]).

Longitudinal study: Based on electrophysiological studies showing a normalisation of certain event-related potentials (ERP) in the days leading up to a migraine attack and/or immediately afterwards [33,34,35,36,37], it was predicted that coherence thresholds would fall in the days leading up to a migraine attack and remain at a lower level immediately afterwards. 

For both studies, there were not expected to be differences between those with migraine with or without aura, consistent with previous psychophysical tests of visual processing [4,5,6,25,26,27,28].

The Group study confirmed that the data obtained from an online study produced comparable results to those obtained under controlled laboratory conditions for Task 1 (vertical coherent motion). The Longitudinal study showed that thresholds for discriminating vertical coherent motion varied with the migraine cycle for a majority of the participants. Performance improved in the two days prior to a migraine attack, and it remained improved for two days afterwards. This outcome points to the possibility of developing sensitive visual tests that patients can use at home themselves to predict an impending migraine attack and so take steps to try to abort it or, if it is inevitable, be able to plan their lives around it.

## 2. Materials and Methods 

### 2.1. Participants

Participants were recruited from an existing migraine panel, advertisements in GP surgeries and hospital neurology clinics, using social media and from an online crowd-sourcing platform (Mechanical Turk). Participants were asked to complete the study on multiple occasions, ideally daily or every other day, over four months. The exclusion criteria were:

- failing to answer the questions in questionnaires
- providing information that was not consistent with a participant classifiable as with or without migraine according to the International Headache Society (IHS) criteria [1]
- reporting poor eyesight in either eye (they were asked whether they could read the text on the back of a compact disc case held at arm’s length, with each eye separately; wearing spectacles or contact lenses was allowed)
- being under 18 years of age
- having a neurological or other condition that could affect visual acuity or day-to-day vision, including photophobia (examples were given: epilepsy, multiple sclerosis, diabetes, lupus, and macular degeneration)
- not completing a test session when migraine- or headache-free for two or more days before that test session and for two or more days afterwards (these migraine- or headache-clear days were used to generate a reference score for each task and each observer to enable group comparisons)

To illustrate criterion (vi): if a participant completed seven test sessions on consecutive days and experienced a migraine on the fourth day, that person would be excluded as they provided only one session (day 1) when it could be established that they were migraine-free for the two days before and the two days after a migraine attack. Day 7 could not be included, as it could not be known whether a migraine may have occurred on days 8 or 9, in which case day 7 would have been within two days of the next attack. That participant would have to complete 9 test sessions to be included and to report no migraine on days 7–9 for their performance on day 7 to be included as that participant’s migraine- and headache-free reference score for the Group study. They would have to complete many more test sessions to be included in the Longitudinal study. For every participant, the first two test sessions were discarded as practice, and the last and penultimate test sessions were excluded as they were unclassifiable with respect to the next migraine or headache attack.

Of the 161 participants who signed up and completed the visual tasks, 118 were excluded due to insufficient data: 107 completed only 1 or 2 sessions in total, 11 were excluded using criteria (vi). A further seven were excluded using criteria (i) to (v), leaving 37 who had completed sufficient test sessions to be able to establish a migraine- and headache-free reference score for each visual task (13 migraine with aura, MA; 12 migraine without aura, MO; 12 control participants, see Table 1). Data from these 37 participants were included in the Group study. Eleven migraine participants completed sufficient test sessions to be able to track performance in relation to migraine proximity. These eleven were included in the Longitudinal study. None took any migraine prophylaxis and one took sumatriptan at attack onset. 

Frequency estimates were obtained by asking the participants to think back over the last three months and to estimate how many migraines (or headaches, for the control group) they had experienced. Then, they were asked to think about whether their migraines or headaches had been more or less frequent than usual over the last few months and use this to estimate how many they had in the last year (after [3]). 

The frequency estimates (Table 1) should be taken as approximate, as consecutive days with migraine may have been counted as separate attacks. For example, 28 reported that their attacks could last for more than a day, particularly when unmedicated. These estimates may also contain counts of headaches that were not migraines, although those with migraine were asked to count migraines only. For these reasons, median frequencies are also included in Table 1. Those who completed multiple sessions and had reported the highest frequencies tended to experience them in mixed bouts, having some attacks with aura and some without over several days, with days clear in-between each bout. This may have complicated their assessment of their migraine frequency and perhaps led to overestimation of the number experienced in 12 months.

All participants had the chance to win a small monetary prize for their participation. Those recruited from the online crowd-sourcing platform (Mechanical Turk) received a small payment upon completion. Informed consent was obtained in accordance with the declaration of Helsinki (1991), and ethical approval was obtained from Birkbeck’s Department of Psychological Sciences ethics committee. Participants had to read background information to the study on the first online page and to manually tick multiple items on a consent checklist in order to proceed.

### 2.2. Stimuli and Procedure

#### 2.2.1. Motion

The motion tasks presented a cloud of dots (random dot kinematograms, RDK) moving either up or down (Task 1) or moving in directions up and 45° to the left, or down and 135° to the right, of vertical (Task 2). On different trials, the ratio of coherently to randomly moving dots was varied to determine coherence thresholds (the proportion of dots needed to move in a coherent direction for motion direction to be reliably identified). Task 1 was adapted from previous research in the author’s laboratory [73]. That study had revealed large group differences for motion moving vertically (up or down): the migraine group needed a higher proportion of coherently moving dots to reliably judge motion direction. 

**Figure 1:**
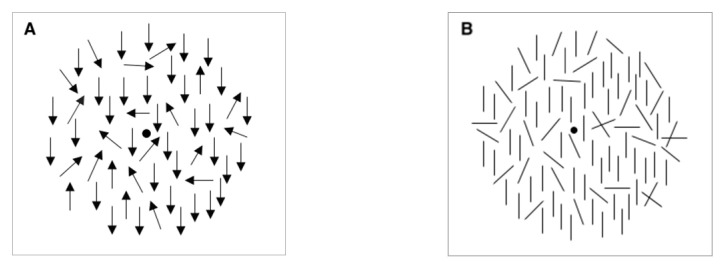
Examples of one of the coherent motion (**A**) and orientation (**B**) tasks. Both depict 60% coherence. A: Each arrow represents the trajectory of a moving dot: 60% are depicted moving downwards, the remainder are depicted moving in random directions (Task 1: is the overall direction of motion up or down?) B: 60% of the lines are oriented vertically, the remainder have random orientations (Task 3: is the overall orientation of the bars vertical or horizontal?) These figures are for illustration: the experimental displays each contained 250 elements.

Task 2 was adapted from previous research showing an OE for coherent motion [69]. That study used a two-interval forced choice procedure: a standard direction of motion was first presented, followed by a comparison display containing motion in a slightly different direction. The task was to decide whether the motion in the comparison display was oriented clockwise or counter-clockwise from the standard. To be consistent with Task 1, in the present study, a single display was used on each trial, and the task was to decide if the overall motion was up and to the left or down and to the right; there was no standard display for comparison (Figure 1A). 

Both coherent motion displays presented 250 randomly positioned black dots (2 pixels by 2 pixels) on a central light grey circular background with a black fixation point presented at the midpoint. The background had a radius of 195 pixels and RGB values of 238. The fixation point remained on screen throughout each block of trials. The dots changed position with a speed of 5°/sec on a 1024 × 768 screen viewed at 45 cm (after [73]). Dots were deleted and repositioned on successive frames if their trajectory would have traversed the outer diameter of the circular background or crossed into the fixation point. 

#### 2.2.2. Oriented Lines

The orientation tasks presented 250 short line segments (2 pixels by 15 pixels) oriented either vertically or horizontally (Task 3) or oriented at 45° to the left or right of vertical (Task 4). Rather than using gratings or virtual lines [65], Tasks 3 and 4 were adapted from Johnston et al. [74]. That study examined orientation discrimination in dyslexia, and here their task was adapted for migraine. Ninety per cent of the lines were initially oriented in a single direction and were interspersed with line segments oriented in random directions (Figure 1B). The proportion of line segments that were oriented coherently was varied on different trials. The short line segments were presented on the grey circular background with the central fixation point. 

### 2.3. Questionnaires

The following questionnaires were presented in the first session:

- A migraine and headache questionnaire to confirm that the participants could be classified as having migraine or not, based on the IHS criteria [1]. Those with migraine were asked whether they experienced symptoms before or during the migraine (visual, pins and needles or numbness, speech difficulties, lack of coordination, dizziness, buzzing in the ears, loss of hearing). They were asked to describe any of the symptoms that they endorsed with a combination of pop-up questions (for visual symptoms and speech difficulties) and text boxes where they could describe their experiences in their own words.
- A list of common migraine and headache triggers that has been used in previous research [4,5,6]; see Table 2. It includes food, beverages, stress (onset or offset), hormonal factors (in women), dehydration, smells, visual stimuli, and an open-ended question where participants can list other visual and/or non-visual migraine or headache triggers. The participants specified whether each item commonly, occasionally or never caused headaches; these were coded as 2, 1, or 0, respectively.
- The Visual Discomfort Scale [75].
- The Cardiff Anomalous Perceptions Scale [76].

Results from the trigger inventory, the Visual Discomfort Scale and the Cardiff Anomalous Perceptions Scale have been submitted elsewhere and are not included here [77]. 

### 2.4. Procedure

This was an online study, so the participants completed the study on their own computers. At the start of the first session, they were presented with the questionnaires, followed by the four visual tasks. On subsequent test sessions, they were presented with a few questions asking whether they had had a migraine or headache since the last test session, and if yes: (i) whether it was a migraine or headache (for those with migraine, control participants were only asked if they had had a headache), (ii) when the migraine or headache had started, and (iii) when it had ended; then followed the four visual tasks. The visual tasks were presented in a random order. The first session took 30–40 min to complete; subsequent sessions were completed in approximately 10 min. 

For all tasks, after each trial, the grey background and fixation point remained on screen until a response was made. All displays were viewed binocularly. Before each block of practice trials, an instruction page was displayed, telling the participant which task was about to be presented and asking them to view the display at a distance of 45 cm. Before the experimental trials, another instruction page appeared that repeated the request to view the display at a distance of 45 cm. Both instruction pages remained on screen until the participant commenced the trials with a keypress. During the motion tasks, 90% of the dots initially moved in a coherent direction, while the remainder moved in random directions. For the orientation tasks, 90% of the line segments were initially oriented in one direction, while the remainder were drawn at random orientations (Figure 1). 

After 16 practice trials at 90% coherence, the proportion of coherently moving dots, or coherently oriented line segments, was either decreased or increased on consecutive trials, depending on whether the preceding response had been correct or incorrect. A beep sounded each time an error was made. The level of difficulty was adjusted to determine the minimum proportion of coherently moving dots, or the minimum proportion of coherently oriented lines, needed to reliably determine their direction of motion, or overall orientation, amongst the remaining elements that were randomly moving dots or randomly oriented line segments. Each trial lasted for 70 msec.

For each task, a two-down, one-up staircase procedure was used to converge upon a threshold coherence corresponding to a 71% correct discrimination rate [78,79]. The two-down, one-up staircase procedure, and the resulting 71% threshold discrimination rate, were chosen to achieve a reasonable estimate of threshold without needing an excessive number of trials. An initial step-size of 10% was reduced to 5% after three staircase reversals and then to 2% if the displayed coherence reached 10% or less. If the coherence level subsequently moved above 10%, the step-size increased again to 5%. Changes in step-size as the staircase progressed were determined following pilot trials designed to determine task difficulty. The three levels were selected to keep the procedure efficient yet capable of using step-sizes appropriate to the level of difficulty experienced by each participant. The staircases terminated after 10 reversals and the average of the last four were taken as the estimate of coherence needed for a correct discrimination rate of 71%.

Each of the motion and orientation tasks used a single-interval, two-alternative forced choice (2AFC) procedure, in which participants were instructed to press one of two of the arrow keys to indicate in which direction the dots were moving coherently, or what the orientation was of the majority of the short line segments. In Task 1, the participants used the up and down arrow keys to indicate that the majority of dots were moving in an upward or downward direction. In Task 2, they used the left or right arrow keys to indicate whether the dots were moving up and to the left or down and to the right, respectively). In Task 3, they used the right or left arrow keys to indicate that the overall orientation of the line segments was to the right or left of vertical, respectively. In Task 4, they used the left or up arrow keys to indicate that the overall orientation of the line segments was horizontal or vertical. 

The order of the tasks was randomised for each participant and each test session.

### 2.5. Statistical Analyses

Statistical analyses for the Group study were conducted using SPSS version 26 (SPSS Inc., Chicago, IL, USA). Descriptive statistics for the Longitudinal study were calculated in Microsoft Excel version 16.

## 3. Results

### 3.1. Group Study

The Group study used the data from a single session for each participant (their reference score for each task). After the first two sessions had been discarded as practice, the reference score was the next session completed when each participant was migraine- or headache-free for at least two days prior to that session and for at least two days afterwards. The reference scores for the four tasks and for each group (migraine with aura, MA; migraine without aura, MO; and control) were normally distributed (Kolmogorov–Smirnov tests, *p* > 0.05), apart from one task (L/R Line segments). This was rectified by the removal of a single outlier. Group differences were assessed with *a priori t*-tests.

**Figure 2:**
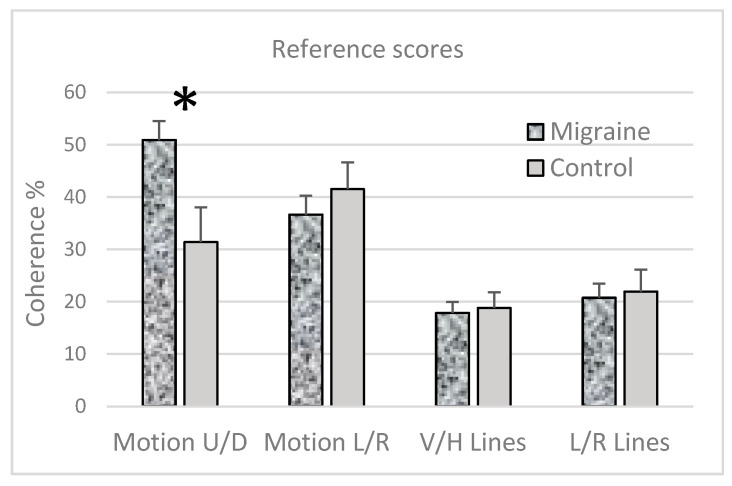
Group study. Coherence thresholds for the four tasks and each group (the MA and MO groups did not differ significantly and were combined into one migraine group). * denotes a significant difference (*p* < 0.01). Error bars are ± 1 standard error. U = motion upwards, D = motion downwards, L = leftwards motion or lines oriented to the left of vertical, R = rightwards motion or lines oriented to the right of vertical, V = vertically oriented lines, H = horizontally oriented lines.

There were no significant differences between the MA and MO groups for any of the tasks; therefore, their data were combined {all *t’s* < 1). The group averages for each task are shown in Figure 2. For the Motion U/D and Motion L/R tasks, coherence refers to the percentage of dots that needed to be moving together in the same direction for participants to be able to reliably discriminate motion with an accuracy of 71% (the remaining dots moved in random directions). For the V/H Line and L/R Line tasks, coherence refers to the percentage of short line segments that needed to be pointing in the same direction to judge the overall orientation in the display with an accuracy of 71% (the remaining lines were oriented at random angles). 

There are clear group differences for Task 1, judging upwards or downwards motion in the random dot displays, but little evidence of group differences for Tasks 2–4. This was confirmed with four *a priori t*-tests: Task 1 (Motion U/D *t*(34) = 2.99, *p* = 0.007); Task 2 (Motion L/R); Task 3 (V/H Lines); and Task 4 (L/R Lines) each *t* < 1, *NS*.

### 3.2. Longitudinal Study.

Eleven migraine participants completed enough test sessions to be able to track several of their migraine cycles. Two completed the tasks almost every day for a period of three or four months (MA1 and MA4 in Table 2). The remainder completed tasks over a couple of months but participated less regularly. Overall, the largest number of sessions was completed between attacks and the fewest in the two days preceding each attack. For several, the frequency of testing declined as the time elapsed since the last migraine increased, until another migraine occurred and testing resumed, resulting in more test sessions for the days following attacks than for the days before. For seven, migraine attacks came in clusters over a period of a few days or for a week or more and were followed by a lengthier period without attacks. Days when testing was completed but the session could not be classified as clearly between attacks, within two days before an attack, or within two days afterwards were excluded from the summaries. Therefore, the remaining data, classified into three phases of the migraine cycle (between, pre, and post attack), were uncontaminated from the residual effects of migraine attacks that occurred mid cluster.

The average data (±1 standard deviation) for each participant in each phase of the migraine cycle are presented in Table 2. Entries where the averages are based on more than three cycles are depicted in bold. Performance in-between attacks was treated as a baseline and performance during the preceding two days and subsequent two days are presented as coherence thresholds and as the percentage change from baseline. Negative changes indicate that performance improved (coherence thresholds fell), and positive changes indicate that performance declined (coherence thresholds increased). 

Cohen’s effect size, d, was calculated for these change scores [80], using the pooled standard deviation of the two samples for each comparison (performance at baseline compared to during the preceding two days, and performance at baseline compared to during the subsequent two days). Effect sizes were corrected to reduce bias in the pooled estimate of the standard deviation [81]. Pooled estimates were used due to the unequal numbers comprising the two groups in each comparison. Cohen [80] proposed effect sizes that could be roughly classified as small (0.2), medium (0.5), and large (0.8). The percentage change in scores, from baseline, that resulted in effect sizes of 0.5 or greater, and that were calculated from more than three cycles, are highlighted in red and in bold in Table 2. The pattern of red, bold entries indicates that there were reliable changes in performance across the migraine cycle for most of the observers. Those without reliable changes had completed the fewest test sessions at one or more stages of the migraine cycle.

The first task, coherence thresholds for vertical dot motion, yielded the most consistent pattern of results. For 9/11 participants, performance improved in the two days prior to an attack and in the two days following an attack, compared to baseline. Performance improved for both of these stages in seven participants. Effect sizes for improved performance were 0.5 or larger for seven participants. Instances of performance decline compared to baseline were small and associated with effect sizes of 0.3 or less.

Coherence thresholds for dots moving up and to the left or down and to the right (Task 2) produced a similar pattern for improvement before or after attacks, but with fewer observers and generally smaller effect sizes. For 5/11, performance improved in the two days prior to an attack and for 9/11 in the two days following it, compared to baseline. Performance improved for both of these stages for four participants. Effect sizes for improved performance were 0.5 or larger for six participants. There was a greater mix of performance improvement and decline, however, compared to Task 1: five participants showed an improvement for one of the sessions and a decline for the other.

There was again a mix of performance improvement and decline for line segments oriented vertically or horizontally (Task 3): three participants showed improvement in performance for both pre- and post-migraine sessions, compared to baseline, two showed a decline in performance for both sessions, and four showed an improvement for one of the sessions and a decline for the other. Some of the changes in performance were small.

Similarly, for the line segments oriented to the left or right of vertical, two participants showed improvement in performance for both pre- and post-migraine sessions, compared to baseline, two showed a decline for both sessions, and five showed an improvement for one of the test sessions and a decline for the other. Again, some of the changes in performance and associated effect sizes were small.

In summary, the motion tasks produced the most consistent pattern of results. Of the two motion tasks, thresholds for discriminating coherent motion in dot displays moving vertically showed more consistent improvements in performance just before and just after a migraine attack, compared to baseline, than the motion task that presented oblique motion directions.

### 3.3. Association with Visual Triggers

It is possible that those migraine participants who show changes in aspects of visual perception associated with the migraine cycle may be those who report visual stimuli to be capable of inducing their migraine attacks. The trigger questionnaire asked whether various visual stimuli could trigger migraine, such as flicker, stripes, alternative light, and shade, or other visual stimuli. Other visual stimuli were reported to be glare, television or cinema, reading, computer (over) use, sun reflecting off chrome or water, and abrupt transitions from light to dark. Participants were asked whether each item commonly, occasionally, or never triggered their migraine, which was coded as 2, 1, or 0. There were no significant correlations between scores for these trigger items and performance on each task, however, nor between the visual trigger items and the percentage change scores (Spearman’s *r_s_*).

## 4. Discussion

This study sought to track changes in performance on four visual tasks over the migraine cycle. As mentioned in the Introduction, there are a growing number of reports that various electrophysiological measures (EEG and ERP) can track migraine periodicity [30,33,34,35,36,37,38], as can imaging the brain using various MRI techniques [46,47,48], but this research has few realistic everyday applications. 

Coherence thresholds were measured for two motion and two orientation tasks. The study was conducted online for pragmatic reasons: it would be unmanageable to ask participants to come into a laboratory on multiple consecutive days over several months, it would be impossible for a researcher to travel with equipment to visit multiple participants regularly, and it would be impractical to give each participant their own device to complete the studies remotely. 

The first part of the study was designed as a check that the data obtained from an online study were sensible—that is, that it produced comparable results to those obtained under controlled laboratory conditions [73]. The task selected was motion coherence for vertically moving random dot displays (Task 1), and the results did indeed show expected group differences when participants with migraine were compared to those without (Figure 2). The proportion of dots that needed to move together coherently in order to judge the overall direction of motion was larger in the migraine than in the control group. These results indicate that the data collected from this online study were as expected, despite the loss of control over the environment in which the test was performed.

### 4.1. Group Comparisons: Motion Coherence

Task 1 was adapted from two earlier studies [73,74]. Johnston et al. [74] reported motion coherence thresholds of 16.8% for males and 21.9% for females for their control group. In the present study, the control group’s average threshold at 31% is somewhat larger, but it is not dissimilar given the different numbers of moving dots (250 versus 200), different motion speeds (5°/sec versus 2.3°/sec), display duration (70 versus 430 msec), average ages of the participants (28 ± 11 versus 22 ± 5) and coherence level that defined the threshold (71% correct discrimination versus 79%). The parameters chosen for the present study were selected to replicate one of the tasks used by Shepherd et al. [73]. The results from Task 1 are similar to those from that earlier study: their motion coherence thresholds were reported to be 59% for their migraine and 41% for their control groups. Here, the migraine and control groups’ average thresholds were 51% and 31%, respectively.

Ditchfield et al. [82] also reported higher motion coherence thresholds in migraine (migraine: 11%, control: 8%). They first established approximate thresholds for each observer (79% correct discrimination) and then selected seven coherence levels based on that estimate, which were used for the main experiment. Therefore, each observer was presented with a different set of coherent motion displays that were each presented once in a single run. Unfortunately, they do not provide the data for all observers nor do they explain how they selected the seven coherence displays for each observer. 

Their reported thresholds are substantially lower than those reported here, which may reflect differences between the two tasks: different number of moving dots (250 versus 100), different motion speeds (5°/sec versus 2.9°/sec), different decisions (was motion up or down versus which of two displays contained downward motion, the other contained random motion); different display durations (70 msec versus 400 msec), different viewing conditions (binocular versus monocular), and different ways of defining threshold (71% versus 79%). Given some uncertainty regarding what was presented to each observer, the only comparison that can be made is their finding of higher motion coherence thresholds in migraine, which mirrors the group differences found in the present study and those reported by Shepherd et al. [73].

Task 2 was included as an alternative motion task using oblique directions. Thresholds would indicate an oblique effect if they were larger for oblique motion than for vertical (Task 1). Figure 2 does show an oblique effect for the control group (Figure 2, compare Motion U/D to Motion L/R, grey bars) but a reverse OE for the migraine group. Pilz and Papadaki [72] have reported a larger OE for motion using RDKs for horizontal/oblique discrimination thresholds compared to vertical/oblique and have also highlighted large individual differences when naïve participants are tested. Dakin et al. [70] have reported little evidence of an OE for motion using RDKs when the coherently moving signal dots were displayed with a broad distribution of noise dot directions compared to a narrow distribution that was close to the motion direction of the signal dots. The former condition is similar to the displays used here with noise dots moving in random directions. Neither study screened for migraine. Future laboratory research could explore motion coherence for additional motion directions to determine how specific the group differences are to vertical and to confirm a reverse oblique effect for motion in migraine. 

### 4.2. Group Comparisons: Orientation Coherence

The two orientation tasks (Tasks 3 and 4) were also novel in migraine research and were selected as orientation discrimination has been shown to be impaired in migraine [65]. In that study, orientation discrimination thresholds were larger in migraine, compared to a control group, only for oblique orientations (45° clockwise from vertical). In the present study, coherence thresholds for the oblique orientations (±45° from vertical) were marginally larger than the thresholds for cardinal orientations for each group (Figure 2, compare V/H Lines to L/R Lines), but the differences were not statistically significant, nor were there any statistically significant group differences. 

Tasks 3 and 4 were also adapted from Johnston et al. [74] and depicted the global form in a multi-element display. They reported the average coherence required to judge global orientation (vertical or horizontal) to be 15.5% for females and 14.8% for males in their control group. These values are close to the 18.8% reported here, which adds weight to the reliability of the results from the present online study despite the lack of an OE. 

Tibber et al. [65] used explicit lines (Gabor patches) and single virtual lines rather than judgments of the overall orientation of multi-element displays. The latter relies on pooling orientation information across the multiple display elements and ignoring elements with random orientations. Crowding of display elements and the presence of irrelevant elements are factors that would influence performance. The former relies on the local processing of single display components. The type of data obtained is also different: the coherence task measures the percentage of elements needed to be oriented in the same direction to give a given accuracy rate (here, 71%); the local tasks measure the angular difference needed to discriminate a comparison display from a standard (again with an accuracy of 71% in [65]). 

The reason for the lack of a global OE in the present study could be explored in further basic research (not related to migraine). This is the first study to use multi-element displays to investigate the OE for orientation. In future research, it would be useful to include the classic OE and have the same participants complete both types of trials. For multi-element displays, display density, display size, and the size of the area that contains signal lines within a larger background could be manipulated to clarify the roles of crowding and spatial pooling [83].

Ditchfield et al. [82] also compared global form perception in migraine and control groups in modified Glass patterns [84]. Glass described global form percepts (concentric circles) in images containing pairs of dots with correlated locations, which were obtained by superimposing one random dot pattern on itself, but slightly rotated, and viewed monocularly. Ditchfied et al. [82] used short line segments instead of correlated dot pairs. They adopted the same procedure as for their assessment of global motion: approximate thresholds were first obtained for each observer (79% correct discrimination); then, seven coherence levels were selected based on that estimated threshold. Therefore, each observer was again presented with a different set of modified Glass displays with different amounts of coherence. Differences in displays, tasks, and definitions of threshold makes formal comparison between the two studies difficult—the only possible comparison is with the overall pattern of results—they reported higher thresholds in their migraine group compared to their control group (migraine: 24%, control: 16%), whereas here, the coherence thresholds for both groups was around 20% for Tasks 3 and 4. It is possible that Tasks 3 and 4 in the present study were too easy to reveal small group differences. Differences may be revealed using a criterion for threshold stricter than 71% correct.

An alternative assessment of local and global processing of static form involves hierarchical stimuli where one overall, or global, shape is comprised of different individual local elements, and the task is to identify ether the global shape or the local elements. For example, a large letter (e.g., H or S) can be made up of smaller, different letters (also either all H or all S) [85]. Typically, the reaction times (RT) to identify either the global letter or the local letters are recorded for trials where the global and local letters are congruent (all Hs or all Ss) or incongruent (if the global letter and local letters differ). Observers are fastest to identify the letters on congruent trials and are typically faster to identify the global letter, rather than the local, for both congruent and incongruent trials, which is referred to as global precedence. 

Koppen et al. [86] have reported no global precedence in migraine either between attacks or at 1 and 2 days after an attack; indeed, the migraine group was marginally slower to respond to the global letters than to the local. They included tasks to assess attention (alerting, orienting, and executive control) and working memory and found no group differences. They suggested that feature integration processes could be disrupted in migraine and speculated that pathways in the visual system tuned to low-spatial frequencies may be impaired. There has been a line of research that has attributed global precedence to processing within pathways tuned to high or low spatial frequencies and/or to activity in the parvocellular and magnocellular pathways in the visual system (reviewed in [87]), despite Navon [85] originally dismissing the idea. Navon reported that performance asymmetries depended on the *relative* size of local and global elements, not their absolute size. Smaller elements were not processed less frequently, nor less accurately, than larger ones, unless they were also a local element in a global shape.

A disruption of feature integration, or the pooling of local elements, should lead to slower RTs for global incongruent displays than for global congruent displays in migraine, whereas RTs to both of these displays should not differ for control participants. Unfortunately, Koppen et al. [86] did not provide the data for the global incongruent trials and global congruent trials; they only provided the results for the global trials combined. Therefore, their data cannot clarify whether the integration of local elements into a coherent global form is impaired in migraine.

### 4.3. Group Comparisons: Local Versus Global Processing

The multi-element displays used in this study (and others) have been described as showing the perception of global motion and global form [73,74,82]. Impaired performance using these displays has been attributed to anomalous processing involving both early and later cortical areas in migraine [73,82]. The anomaly has been attributed to various models of cortical function, although the precise nature and practical consequences of the anomaly are seldom made explicit [23]. For example, cortical hyperexcitability in migraine has been discussed for over 30 years and is usually attributed to a lack of inhibition [3,88,89]. A lack of cortical excitation has also been discussed; however [64,66,90,91], so have hybrid models combining elements of both hypoexcitability and hyperexcitability, e.g., low cortical pre-activation combined with heightened responsiveness [64,66,91,92,93,94,95].

When models are poorly defined, it is possible to make a model fit any pattern of data [23]. For example, a model of hyperexcitability that results in an increase in background neural noise, against which weak threshold signals must be detected, would reduce the signal-to-noise ratio in a migraine, compared to a control group, and impair performance [82,96]. Alternatively, a model of hypoexcitability that results in a smaller response to incoming signals against a normal level of background noise would also lower the signal-to-noise ratio and predict poorer performance in migraine. On the other hand, if both background noise and the neural response to incoming signals are increased or decreased together, the signal-to-noise ratio and would be maintained and result in no group differences. The hybrid hypo–hyperexcitable model combining low pre-activation with a potentiation of responses to incoming signals could result in the cortex being highly reactive, or having a less selective response to incoming signals, which would decrease the signal-to-noise ratio and so predict impaired detection and discrimination. 

Further elaboration of the arguments for a general model of cortical function in migraine can be found in [23,89,97,98]. It has also been suggested that one general model of neurological function in migraine may be inappropriate, as different stages within the visual pathways may be affected in distinct ways and that patterns of group differences, consistent with hyperexcitability versus hypoexcitability, emerge depending on the particular conditions or tasks employed [27,66,99,100]. Rather than a single type of dysfunction throughout a cortical area or areas, it may be more appropriate to consider cortical dysfunctions in migraine, the nature of which depends on the neural circuits driven by each task. For an early review, see [23]; for later discussions, see [25,26,29,73]. 

For threshold tasks, all of the models refer to background noise against which the relevant signal (motion direction or orientation) must be detected or discriminated, yet the question of what noise is, and how to quantify it, is relatively new in migraine research. General models of cortical hyperexcitability or hypoexcitability refer to internal or endogenous neural noise, be it background neuronal chatter uncorrelated with any visual input, or variability in response to relevant stimuli. The global motion and form displays present the visual system with additional external noise, that is, variability in the displays themselves. Attempts to dissociate effects of internal and external noise on performance have recently provided evidence for normal levels of internal noise in migraine for judgments of motion direction, orientation, and size [101]. 

One way to distinguish effects of internal and external noise is by manipulating the type of external noise presented in each display. In equivalent noise (EN) paradigms, the noise is correlated with the global motion direction or orientation of interest (various degrees of noise can be presented based on multiples of the standard deviation of the distribution of motion directions or orientations in each display), whereas in coherence paradigms, the motion direction or orientation of the noise elements is random. Using an EN analysis, Tibber et al. [101] concluded that elevated coherence thresholds in migraine occur due to an inability to exclude irrelevant external noise, not because of impaired local and global processing, nor an inability to integrate local motion or orientation signals into a global percept (cf. [102]). That is, local and global processing is normal, as is the ability to integrate local elements. Impaired performance for coherence tasks occurs when signal and *external* noise must be segregated prior to the integration stage. This conclusion is inconsistent with any general model of cortical hyperexcitability or hypoexcitability that relies on abnormal baseline (*internal*) neuronal firing rates. 

The tasks in the present study were selected from the literature as likely to show group differences and were amenable to being completed online; they were not selected to test a particular model of cortical processing in migraine *per se*. It would be useful to include EN tasks as well as motion coherence in future studies to track the migraine cycle.

Tibber et al. [101] speculated that attention may be involved in the segregation of external noise from the coherent elements in threshold detection and discrimination tasks. When external noise is correlated with one direction of motion or orientation (in EN displays), the optimum strategy is to group or pool the response to all of the elements, as every element contributes to the overall motion direction or orientation. When the external noise is uncorrelated with the overall motion direction or orientation, the coherent signal and noise dots (or lines) compete for selection and interfere with effective grouping of similar items: the noise elements dilute the global signal and disrupt the visual system’s ability to track or extract the coherent elements [70,71]. A failure in endogenous attentional selection in migraine could account for impaired coherence thresholds despite normal levels of internal noise at local and global motion sites [101].

There are a number of studies that have investigated cortical function in migraine and have branched away from purely perceptual paradigms to include cognition and attention. There have been many electrophysiological studies using ERPs during attentionally demanding tasks, including contingent negative variation and auditory and visual odd-ball tasks. Other studies have used neuropsychological test batteries to assess general cognitive function (for an early review, see [103]). Recently, there has been renewed interest in tests of attentional and cognitive function in migraine due, in part, to the growing number of reports of widespread functional and structural alterations in brain areas involved in visual attention, visual spatial memory, somatosensory and somatomotor processing, including temporal, frontoparietal cortical areas, and the cerebellum [54,104,105,106,107] (see also Introduction). A recent review of attention and neuropsychological function in migraine, however, reported trivial or inconsistent effects for tests of processing speed, attention, verbal memory, verbal skills, working memory, sustained attention, and inhibition [108]. They describe mixed results regarding deficits in visual memory, motor dexterity, visuospatial/constructional skills, visual reasoning, and mental flexibility. They concluded that the evidence for an effect of migraine in any cognitive domain is weak. The present results are compatible with this conclusion, as a general impairment in cognitive function or in the allocation of attention would lead to impairment across the four tasks, whereas significant group differences occurred only for Task 1. Many of the standard neuropsychological tests are fairly blunt instruments, however, and the review excluded studies on visual processing. Future research could examine aspects of selection and visual attention specifically.

### 4.4. Longitudinal Study

The longitudinal part of the study showed that thresholds for discriminating vertical coherent motion varied with the migraine cycle for a majority of the participants. Performance improved in the two days prior to a migraine attack and remained improved for two days afterwards. Improvement is consistent with results from some of the electrophysiological research that has shown a normalisation of certain ERPs in the days leading up to a migraine attack and immediately afterwards [33,34,36]. Only a few of the recent imaging studies look at activity and/or functional connectivity both immediately before and immediately after an attack: many focus just on the run up to the attack and compare that to measurements made between attacks, or they compare interictal measurements in migraine and healthy volunteers. Nevertheless, the results from the present study are consistent with imaging studies that have shown fluctuations in activity in diverse areas including the brainstem, thalamus, and various cortical areas in the 24 h prior to an attack (see Introduction). It is not suggested that there is a precise connection between the cyclical variations in the measurements from different electrophysiological, imaging, or psychophysical paradigms; rather, the important observation is that three different techniques can each show systematic changes with the migraine cycle. Determining specific links between the results from different paradigms is an avenue for future research.

The lack of significant associations between performance at each stage of the migraine cycle and reports of visual triggers may reflect the small sample size. Eight of the 11 migraine participants reported visual triggers; only three did not (MO1, MO2, and MA4 in Table 2). MO1 and MO2 showed small and inconsistent changes in the 2 days prior and following a migraine attack compared to baseline, although MA4 showed reliable changes for both. Associations between performance at each stage of the cycle and visual migraine triggers may emerge with a larger sample.

The present study used a two-down, one-up staircase procedure, whereby the task was made more difficult following two successive correct responses, and it was easier after every error. This is a common procedure, as it tracks a reasonably high threshold rate (71% correct discrimination [78]) and does not require a large number of trials. One problem with staircase procedures is that if the participant experiences a moment of inattention or distraction and makes multiple errors in a row, the task difficulty can be pushed far from the true threshold value and it can take some time for task difficulty to return to near threshold. A three-down, two-up procedure could be used instead to keep task difficulty close as the threshold is approached. This would increase the number of trials required, but time could be recouped by reducing the number of tasks and/or by reducing the number of reversals before the task terminated. In the present study, the task terminated after 10 staircase reversals, and the average of the last four reversals was taken as the threshold. The number of reversals required to terminate the task could be reduced to eight instead of 10. Estimating thresholds with fewer than eight reversals is risky, as staircase procedures typically take some time to converge and give reliable values: ideally, the last four reversals should all involve similar coherence values. 

There were no consistent changes in performance for the orientation tasks (Tasks 3 and 4) over the migraine cycle: some thresholds fell, some rose for different participants just prior to an attack, or just following one, compared to the migraine-free interval. As mentioned, it is possible that Tasks 3 and 4 in the present study were too easy to reveal small group differences. Differences may be revealed using a criterion for threshold stricter than 71% correct.

McKendrick et al. [41] have also conducted a study to investigate whether performance on visual tasks can be used to predict an impending migraine attack. One task involved luminance increments superimposed on a background of spatial luminance noise; the other assessed a simultaneous contrast effect referred to as centre-surround contrast suppression. 

For the luminance increment task, threshold was defined as the increment needed to reach a 79% correct detection rate. Thresholds were lower for their migraine group than their control group, which was an unexpected result, but they did not vary in the days leading up to or following a migraine attack. 

Centre-surround contrast suppression did, however, change in proximity to a migraine attack. McKendrick et al. [41] used low spatial frequency vertically oriented gratings that slowly drifted to the left or right. The target was a low contrast central grating (40% contrast) embedded in a higher contrast surround (95% contrast). The contrast of a separate grating could be varied to match the central target grating. The observers’ task was to judge which of the two gratings had the higher contrast. Curiously, they now defined threshold as a 50% correct detection rate rather than the 79% adopted for their other study. Thus, threshold is equivalent to chance performance rather than as a threshold that yields consistent results on a majority of trials. Between attacks, they reported that their migraine observers performed at chance when the match grating displayed around 15% contrast, whereas their control group set contrasts nearer to 20%. 

McKendrick et al. [41] tested their participants daily, beginning with days that were headache-free and continuing through a day/days with migraine and then for a week thereafter. They thereby captured only one migraine episode for most of their participants. They reported that, on average, performance varied with the migraine cycle: two days before an attack, contrast suppression decreased (the average group contrast was higher), it remained somewhat decreased one day before an attack, and then it increased during the attack (the average group contrast was lower, and it was lower than on headache-free days). One day after the attack, the average group contrasts increased slightly, by two days after the attack the contrasts set were equivalent to those set between attacks. 

Pairwise comparisons revealed the only significant difference to be between the average decrease in suppression two days before the attack compared to the average increased suppression during the attack, although no mention is made of correction for multiple comparisons. They also report that the majority of observers showed the least suppression (i.e., set the highest contrasts) pre- or immediately post-migraine; however, the figure shows instead that the least suppression occurred for the majority of observers in the days preceding the attack only (N = 5 for two days before the attack, N = 5 for one day before the attack, N = 4 for the day of an attack, N = 1 for one day following an attack, and N = 3 two days following an attack. Outliers are evident in their results but not discussed, nor are the data for individual participants provided. The effects are small at best, and the average contrasts set *between* attacks did not differ significantly to those set immediately before, during, or immediately after an attack. Therefore, centre-surround contrast suppression measured *between* attacks is not a measure that could be used as an indicator of the likelihood of the next attack, as is suggested. They note that interictal differences in centre-surround contrast suppression occurs only for drifting, not static, gratings, which implicates the involvement of areas of the cortex responsive to motion perception. Insofar as their contrast suppression task reflects motion processing, a reduction in suppression prior to an attack is consistent with the data reported in the present study where the coherence required to judge vertical motion direction decreased prior to an attack (Task 1). 

To conclude, in the present study, a simple, non-invasive visual task was sensitive enough to be able to track the migraine cycle in 9/11 of the migraine participants, and the outcome was as expected from the trends shown in some of the earlier electrophysiological and imaging research. Future research should replicate and refine this result. First, the test session could be shortened so that more participants are motivated to continue with daily testing. In the present study, it took approximately 10 min to complete the four visual tasks. There were fewest test sessions recorded for the days immediately prior to a migraine: some participants stopped testing themselves as the number of days from the last attack increased and only started again when they had a subsequent attack, so the data from the preceding two days are missing. Emails were sent to participants to remind them to continue to test themselves regularly, but this was clearly not effective for some participants. A shorter test session, together with email or text reminders, may increase the rate of repeat testing.

It is useful to have a group comparison to check that the results are sensible, as was done here with the reference score for migraine and control groups (Figure 2). In future research, multiple reference scores could be extracted over a period of weeks so that the effects of practice could be assessed in each group. This was not possible in the present study, as the control participants tended to discontinue testing after a few sessions. Again, shorter test sessions and explicit reminders may motivate all participants to continue with the test sessions for a longer period of time.

Migraine is usually an episodic, cyclical condition. Some patients can identify a prodromal phase in the hours or day before the headache when they experience symptoms such as yawning, food cravings, mood swings, irritability or gastrointestinal symptoms [1,21,23], or more general premonitory symptoms including anxiety, tiredness, language and/or reading difficulties, and difficulty concentrating [109]. Others do not have such warning signs and, as mentioned in the Introduction, a common complaint is that their migraine appears unexpectedly. The average attack is over within 24 h, but people can still feel affected or sluggish the next day. Consequently, they live with the anxiety that another attack is imminent that may affect them for up to three days, which can affect their everyday life and have an impact on their social, family, and work activities. For these patients, being able to predict an impending attack would help address that anxiety as they could try to abort it, or, if it is inevitable, be able to plan their life around it. 

The cost of migraine is substantial due to the high prevalence. Prevalence rates are inevitably estimates, as many do not seek medical advice, particularly when there is a family history of headache or migraine. Consequently, the World Health Organisation’s (WHO) report that migraine affects one in nine is likely to underestimate the actual prevalence rates (11% overall, 6–8% males, 15–18% females, which are estimates based on European and American epidemiological studies in 2004) [110]. Estimates from the Migraine Trust (MT) are one in seven [111]. The WHO has a metric, the number of years lost to disability (YLD), which attempts to estimate the years of life lived in less than full health [112]. The WHO list migraine as the 20th most disabling lifetime condition (9th for women), which is based on the number of YLD. Another report cites headache disorders more generally as the 9th most disabling condition, and the 5th for women. In the UK, it is estimated that 90,000 people cannot attend work or education each day because of headaches, amounting to 25 million workdays or schooldays lost every year. The MT [111] estimates the cost at £2.5 billion a year. Evidently, a test that can be used to help patients manage their migraine by tracking their migraine cycle could benefit not only the individual, but the wider community as well.

### 4.5. Overview

This preliminary study has shown that visual tasks can be developed that are sensitive enough to show changes in performance with the migraine cycle, which could benefit the millions of people with migraine. If they can learn to recognise their pattern of performance, they can predict when an attack is likely and take evasive action to abort it (either behaviourally—resting, taking an early night, avoiding other triggers—or by taking appropriate medication early) or plan around it. At the moment, the longitudinal study is based on a relatively small number of migraine participants (four with aura, seven without), so more participants need to be recruited to confirm the results reported here. Suggestions have been made to refine the procedure in future research, as the next step in the process of developing a simple, non-invasive test that patients can use at home. 

## Figures and Tables

**Table 1:** Participant details (means ± one standard deviation and range of scores in parentheses) for age, migraine duration, and migraine frequency. MA: migraine with aura; MO: migraine without aura; C: control group; F: female; M: male; md = median.

Group (Years)	Age (Years)	Migraine Duration (Years Experienced)	Migraine Frequency (Per Year), Median
**A: Group study**			
**Migraine (22 F, 3 M)**	**40 ± 11** **(19–62)**	**23 ± 6** **(5–28)**	**26 ± 17** **(2–60), md = 25**
MA (10 F, 3 M)	41 ± 11 (27–62)	22 ± 7 (5–28)	21 ± 19 (2–60), md = 10
MO (12 F, O M)	39 ± 12 (19–56)	25 ± 5 (12–25)	30 ± 16 (2–48), md = 40
**Control (6 F, 6 M)**	**28 ± 11** **(18–51)**		
**B: Longitudinal study**			
**Migraine (9 F, 2 M)**	**43 ± 11** **(26–60)**	**22 ± 5** **(12–25)**	**32 ± 15** **(5–48), md = 40**
MA (2 F, 2 M)	44 ± 14 (27–60)	20 ± 2 (17–20)	19 ± 17 (5–40), md = 15
MO (7 F, 0 M)	42 ± 10 (26–56)	23 ± 6 (12–25)	39 ± 9, md = 40 (20–48)

**Table 2:** Coherence thresholds for each task and each participant at three stages of the migraine cycle (between migraine attacks, within two days prior to an attack, and within two days following it). MA: migraine with aura, MO: migraine without aura. Columns 3, 6, 9, and 12 show average thresholds (± 1 standard deviation) calculated from the number of sessions completed for each stage (column 2). Pooled standard deviations are presented in parentheses for the calculations of effect sizes [80,81]. “Change” columns show the percentage change in performance for the prior and post thresholds, relative to each participant’s threshold in-between attacks. Figures in bold highlight data derived from more than three migraine cycles. Figures in bold and red show changes associated with effect sizes of 0.5 or larger for those entries derived from more than three migraine cycles.

		Motion V/H			Motion L/R			Lines V/H			Lines L/R		
	# sessions	Av ± 1sd (pooled sd)	change	effect size	Av ± 1sd (pooled sd)	change	effect size	Av ± 1sd (pooled sd)	change	effect size	Av ± 1sd (pooled sd)	change	effect size
**MA1**													
**Interictal**	**24**	**30 ± 11**			**30 ± 19**			**24 ± 11**			**23 ± 10**		
**2 days prior**	**10**	**22 ± 8 (10)**	**−26%**	**0.7**	**30 ± 18 (19)**	**2%**	**0**	**18 ± 9 (10)**	**−22%**	**0.5**	**18 ± 8 (10)**	**−22%**	**0.5**
**2 days post**	**13**	**12 ± 12 (11)**	**−32%**	**0.8**	**26 ± 10 (17)**	**–12%**	**0.2**	**21 ± 10 (11)**	**–10%**	**0.2**	**23 ± 9 (10)**	**–1%**	**0**
**MA2**													
**Interictal**	**10**	**22 ± 21**			**26 ± 20**			**12 ± 5**			**12 ± 3**		
**2 days prior**	**5**	**16 ± 6 (18)**	**–27%**	**0.3**	**18 ± 13 (19)**	**–27%**	**0.4**	**9 ± 3 (4)**	**−27%**	**0.7**	**13 ± 6 (4)**	**9%**	**0.2**
**2 days post**	**8**	**16 ± 6 (17)**	**–25%**	**0.3**	**15 ± 8 (16)**	**−25%**	**0.7**	**13 ± 7 (6)**	**11%**	**0.2**	**14 ± 8 (6)**	**17%**	**0.3**
**MA3**													
**Interictal**	**18**	**66 ± 12**			**37 ± 13**			**18 ± 8**			**13 ± 6**		
2 days prior	2	64 ± 7 (12)	−4%	0.2	48 ± 28 (15)	28%	0.7	22 ± 3 (8)	26%	0.6	10 ± 1 (6)	−27%	0.6
2 days post	3	70 ± 6 (12)	6%	0.3	25 ± 7 (13)	−34%	0.9	21 ± 2 (8)	17%	0.4	21 ± 8 (6)	58%	1.2
**MA4**													
**Interictal**	**22**	**36 ± 18**			**31 ± 14**			**12 ± 6**			**14 ± 9**		
**2 days prior**	**20**	**22 ± 11 (15)**	**−38%**	**0.9**	**25 ± 10 (12)**	**−19%**	**0.5**	**15 ± 6 (6)**	**23%**	**0.4**	**14 ± 6 (8)**	**–1%**	**0**
**2 days post**	**29**	**24 ± 15 (16)**	**−33%**	**0.7**	**26 ± 14 (14)**	**–16%**	**0.3**	**15 ± 5 (6)**	**28%**	**0.6**	**15 ± 7 (8)**	**5%**	**0.1**
**MO1**													
**Interictal**	**10**	**51 ± 18**			**35 ± 12**			**12 ± 5**			**16 ± 7**		
2 days prior	2	47 ± 12 (18)	−8%	0.2	39 ± 9 (12)	10%	0.3	11 ± 1 (5)	−11%	0.3	26 ± 26 (11)	69%	0.9
**2 days post**	**5**	**47 ± 31 (23)**	**–7%**	**0.2**	**32 ± 13 (13)**	**–10%**	**0.3**	**18 ± 11 (8)**	**48%**	**0.7**	**17 ± 6 (7)**	**12%**	**0.3**
**MO2**													
**Interictal**	**9**	**46 ± 11**			**26 ± 15**			**15 ± 5**			**18 ± 9**		
2 days prior	3	50 ± 11 (11)	8%	0.3	38 ± 11 (15)	49%	0.8	13 ± 5 (5)	−16%	0.4	20 ± 5 (8)	8%	0.2
**2 days post**	**8**	**44 ± 15 (13)**	**–3%**	**0.1**	**30 ± 5 (12)**	**17%**	**0.4**	**17 ± 6 (6)**	**8%**	**0.2**	**15 ± 4 (7)**	**−19%**	**0.5**
**MO3**													
**Interictal**	**9**	**55 ± 15**			**58 ± 16**			**17 ± 9**			**12 ± 5**		
2 days prior	0	−	−	−	−	−	−	−	−	−	−	−	−
**2 days post**	**4**	**37 ± 17 (16)**	**−33%**	**1**	**52 ± 13 (15)**	**–11%**	**0.4**	**9 ± 4 (8)**	**−46%**	**0.9**	**11 ± 3 (4)**	**–10%**	**0.3**
**MO4**													
**Interictal**	**13**	**25 ± 12**			**26 ± 14**			**13 ± 13**			**12 ± 6**		
2 days prior	1	23	−8%	−	29	12%	−	12	−7%	−	12	0	−
**2 days post**	**7**	**15 ± 4 (10)**	**−42%**	**1**	**21 ± 8 (13)**	**–20%**	**0.4**	**7 ± 3 (11)**	**−45%**	**0.5**	**13 ± 7 (6)**	**9%**	**0.2**
**MO5**													
**Interictal**	**14**	**30 ± 18**			**32 ± 15**			**18 ± 6**			**16 ± 10**		
**2 days prior**	**4**	**20 ± 15 (18)**	**−32%**	**0.5**	**17 ± 10 (14)**	**−46%**	**1**	**17 ± 6 (6)**	**–3%**	**0.1**	**13 ± 5 (9)**	**–18%**	**0.3**
**2 days post**	**11**	**28 ± 12 (16)**	**–7%**	**0.1**	**31 ± 16 (16)**	**–5%**	**0.1**	**16 ± 9 (8)**	**–9%**	**0.2**	**18 ± 9 (10)**	**9%**	**0.1**
**MO6**													
**Interictal**	**5**	**27 ± 15**			**28 ± 7**			**22 ± 8**			**24 ± 11**		
2 days prior	2	17 ± 16 (16)	−36%	0.5	16 ± 0.4 (6)	−44%	1.6	24 ± 2 (7)	5%	0.1	24 ± 1 (10)	1%	0
**2 days post**	**15**	**17 ± 9 (11)**	**−36%**	**0.8**	**25 ± 14 (13)**	**–9%**	**0.2**	**17 ± 7 (7)**	**−23%**	**0.7**	**17 ± 8 (9)**	**−28%**	**0.8**
**MO7**													
**Interictal**	**40**	**17 ± 10**			**16 ± 8**			**9 ± 3**			**9 ± 3**		
2 days prior	3	8 ± 0.3 (9)	−50%	0.9	15 ± 10 (8)	−7%	0.1	9 ± 4 (3)	0%	0	9 ± 5 (3)	−2%	0.1
**2 days post**	**9**	**20 ± 12 (10)**	**16%**	**0.3**	**17 ± 9 (8)**	**6%**	**0.1**	**11 ± 3 (3)**	**23%**	**0.7**	**8 ± 2 (3)**	**–12%**	**0.4**
